# Discovery of a New World ladybird beetle *Nephaspis indus* Gordon, 1996 (Coleoptera: Coccinellidae: Scymnini) on the Island of Taiwan

**DOI:** 10.3897/BDJ.4.e10537

**Published:** 2016-11-17

**Authors:** Xiaosheng Chen, Xiufeng Xie, Shunxiang Ren, Xingmin Wang

**Affiliations:** ‡ College of Forestry and Landscape Architecture, South China Agricultural University; Guangdong Key Laboratory for Innovative Development and Utilization of Forest Plant Germplasm, Guangzhou, China; § Key Laboratory of Bio-Pesticide Innovation and Application of Guangdong Province, Guangzhou, China; | Guangdong Agriculture Industry Business Polytechnic College, Guangzhou, China

**Keywords:** Coccinelloidea, taxonomy, morphology, spiralling whitefly predator, biological control

## Abstract

**Background:**

*Nephaspis indus* Gordon, 1996 was imported into Taiwan from Hawaii in 1990 as a biological control agent for the spiralling whitefly, *Aleurodicus dispersus* Russell, 1965 (Hemiptera: Aleyrodidae). However, its establishment was not known prior to this study.

**New information:**

*Nephaspis indus* Gordon, 1996, a natural enemy of *Aleurodicus dispersus* Russell (Hemiptera: Aleyrodidae) native to the Neotropical region, is recorded as established in Taiwan for the first time. The present paper provides a detailed further description and illustrations of the adult. Diagnostic characters for the genus and species are given and the nomenclature of this species is also discussed.

## Introduction

*Nephaspis* Casey, 1899 (Coleoptera: Coccinellidae: Scymnini) is a New World genus and currently includes 43 species distributed from southern United States to Argentina (Gordon 1996​). This genus was initially described by Casey (1899) based on two species, *N. gorhami* and *N. brunnea* from Central America. Later, he placed *N. brunnea* as a synonym of *N. gorhami* (Casey 1905). Wingo (1952) described another species, *N. amnicola* from North America. However Gordon (1985) synonymised this species with *N. oculatus* (Blatchley) which was transferred from *Scymnus* to *Nephaspis*, based on the examination of their holotype. *Nephaspis* was first revised by Gordon (1972) who described an additional species *N. cocois* from Brazil and also recognised that the Guyana species *Clitostethus dispar* Sicard, 1929 (Sicard 1929) is congeneric. Subsequently, Gordon (1978, 1982, 1990) described five species from Trinidad, Colombia, Argentina and Puerto Rico and Duverger (1986) described one more species from Nicaragua. In his comprehensive revision of the genus *Nephaspis*, Gordon (1996) recognised and described 32 more species to this genus from South America, transferred *Scymnus convexus* Nunenmacher, 1937 to *Nephaspis* and provided a detailed description of this species.

Members of the genus *Nephaspis* are predators of whiteflies (Hemiptera: Aleyrodidae) (Gordon 1996). In 1988, the spiralling whitefly, *Aleurodicus dispersus* Russell, 1965 which is native to the Central America and Caribbean regions, was discovered in southern Taiwan and soon became a serious pest for fruit trees, vegetables, food crops, shade trees and landscape ornamentals (Chien et al. 2002). Due to the wide range and scattered distribution of its host plant and the difficulty with insecticide spraying on tall trees, biological agents were sought to control this invasive pest. Therefore, *Nephaspis indus* Gordon was imported into Taiwan from Hawaii in 1990 with a view to controlling the spiralling whitefly, *Aleurodicus dispersus* (Wen 1995). However, it is uncertain whether it was established at that time (Chien et al. 2002, Yu 2011).

In this paper, *Nephaspis indus* Gordon, 1996 is recorded as being established in Taiwan for the first time. The detailed further description and illustrations of the adult are provided. Diagnostic characters for the genus and species are given and the nomenclature of this species is also discussed.

## Materials and methods

Specimens examined were collected from the Island of Taiwan and deposited in the Department of Entomology, South China Agricultural University, Guangzhou, China (SCAU). The morphological terms follow Ślipiński (2007) and Ślipiński and Tomaszewska (2010).

Measurements were taken using a micrometer attached to a SteREO Discovery V20 dissecting stereoscope and are defined as follows: (TW) total width, across both elytra at widest part; (TH) total height, at highest part of elytra in lateral view; (TL) total length, from apical margin of clypeus to apex of elytra; (PL) pronotal length, from the middle of anterior margin to the base of pronotum; (PW) pronotal width at widest part; (EW) elytral width, equal to TW; (EL) elytral length, along suture from base to apex including scutellum; (HW) head width, at widest part including eyes.

Male and female genitalia were dissected, cleared in a 10% solution of NaOH by boiling for several minutes and placed on slides for further study. Photographs of the whole beetles and their genitalia were taken using digital cameras (AxioCamHRc and Coolsnap-Procf& CRI Micro*Color) attached to the microscope. The final plates were laid out with Adobe Photoshop CS 8.0.

## Taxon treatments

### Nephaspi﻿s﻿

Casey, 18﻿99

Nephaspis Casey, 1899: 168. Type species: *Nephaspis gorhami* Casey, 1899, by subsequent designation of Gordon 1972: 148.
Nephasis : Korschefsky 1931: 168; Blackwelder 1945: 445 (misspelling).
Nephaspi﻿s﻿ Nephaspis gorhami Casey, 1899Gordon 1972

#### Description

Body elongate oval, moderately convex, with dense pubescence, widest around middle of elytra.

**Figure 1.**
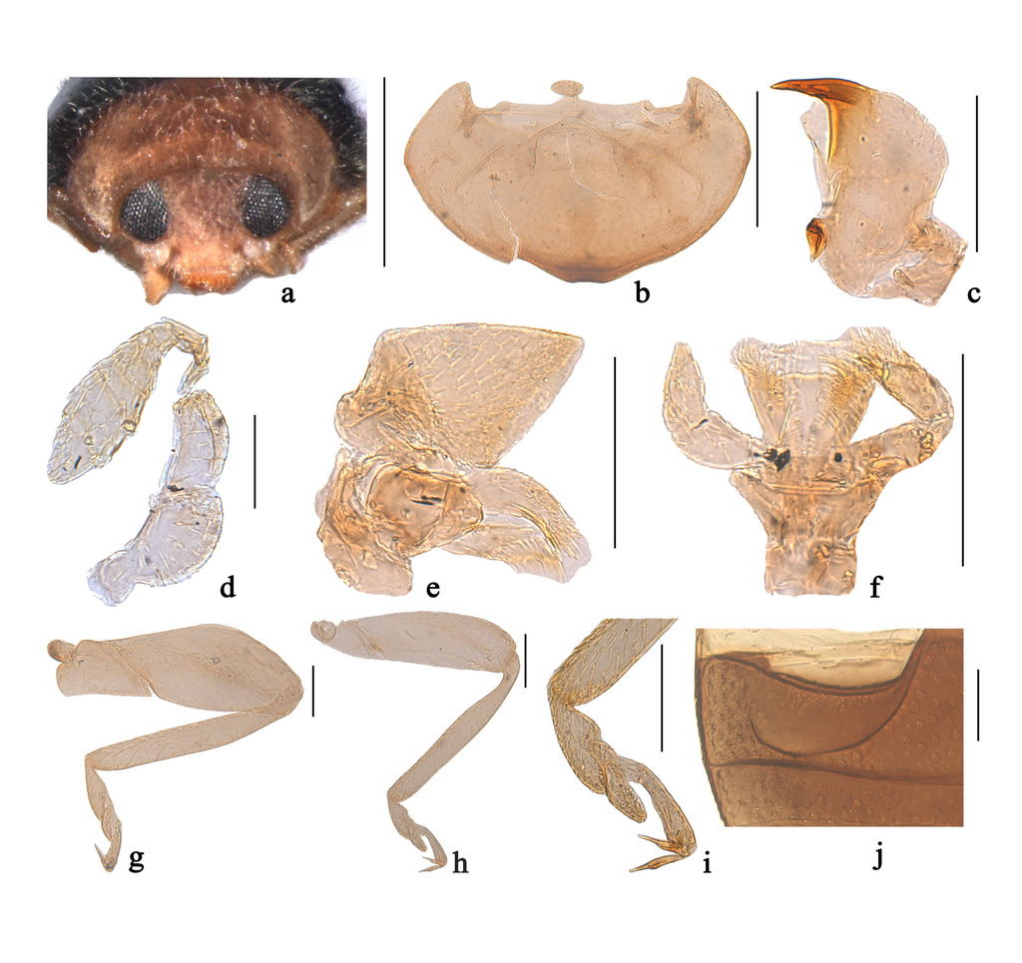
Figure 1. Main characters of the genus *Nephaspis* Casey: (a) head; (b) prothorax, ventral; (c) mandible; (d) antenna; (e) maxilla; (f) labium; (g) hind leg; (h) front leg; (i) tarsi; (j) part of abdominal ventrite 1 and 2. Scale bars: a =0.5 mm, b =0.2 mm, c–j =0.1 mm.

Head with mouthparts directed posteroventrally in repose (Fig. 1a), concealing prosternum; frons wide (Fig. 1a), clypeus extended beyond eye, truncate anteriorly with large, apically rounded flange. Eyes large, finely faceted, inner ocular margin slightly arcuate. Antennae composed of 11 antennomeres (Fig. 1d); 1st antennomere strongly enlarged, curved and distinctly rounded on inner side; 2nd shorter and narrower than the 1st; 3rd obviously smaller than the 2nd; 4th to 6th as wide as 3rd; 7th to 11th forming a fusiform club (Fig. 1d). Labrum exposed, transverse, feebly emarginate anteriorly (Fig. 1a). Mandible with single apical tooth (Fig. 1c). Terminal maxillary palpomere stout, moderately securiform, apical margin strongly obliquely truncate (Fig. 1e). Labial palps with 3 palpomeres, terminal palpomere blunt, subcylindrical (Fig. 1f).

**Figure 2.**
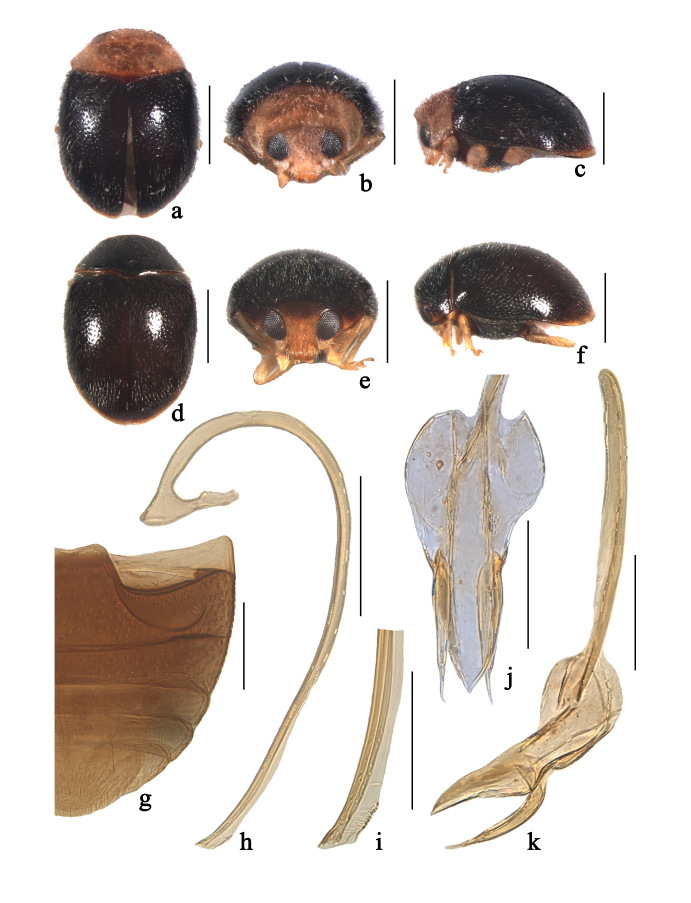
Figure 2. *Nephaspis indus* Gordon, 1996: (a–c) male; (d–f) female; (a, d) dorsal view; (b, e) frontal view; (c, f) lateral view; (g) abdomen; (h) penis; (i) apex of penis; (j) tegmen, ventral view; (k) tegmen, lateral view. Scale bars: a–f = 0.5 mm, g–h = 0.2 mm, i–k = 0.1 mm.

Pronotum convex, hind margin wider than anterior one (Fig. 2b, Fig. 2e). Pronotal hypomeron broad without delimited foveae (Fig. 1b). Prosternum short, straplike, slightly longer than anterior coxa, prosternal process very short, transversely oval (Fig. 1b); prosternal carinae narrowly separated, distinctly convergent anteriorly. Scutellum triangular (Fig. 2a). Elytra slightly wider than pronotum at base, surface finely punctate. Elytral epipleuron narrow and nearly horizontal, inner carina apically incomplete, reaching up to 1st abdominal ventrite. Mesoventrite short, tumid medially, anterior margin truncate, intercoxal area with hind margin deeply emarginate. Metaventrite strongly tumid, anterior margin distinctly ridged, median area strongly prominent forward. Abdomen with six ventrites (Fig. 2g). Abdominal postcoxal lines incomplete (Fig. 1j, Fig. 2g). Front leg slender (Fig. 1h), femora of hind leg distinctly enlarged (Fig. 1g); tibiae without apical spur; tarsi with 4 tarsomeres, claws simple without basal teeth (Fig. 1i).

#### Diagnosis

*Nephaspis* Casey is similar to the Old World genus *Clitostethus* Weise, 1885 in general appearance and shares the following characters with the latter: body small, length less than 2 mm; antennae composed of 11 antennomeres (Fig. 1d); prosternum straplike, prosternal process short, transversely oval (Fig. 1b); abdominal postcoxal line incomplete (Fig. 1j, Fig. 2g); abdomen with six ventrites (Fig. 2g). *Nephaspis* is separated from *Clitostethus* mainly by the tarsi with 4 tarsomeres (Fig. 1i) and the stout basal antennomere (Fig. 1d). However, *Clitostethus* has the tarsi with 3 tarsomeres and the slender basal antennomere.

#### Distribution

This genus is apparently endemic to Neotropical region with a natural geographic range extending from southern United States (Florida, Louisiana and Texas) and Mexico to Argentina (Gordon 1996). In addition, several species of this genus are specialist predators of the spiralling whitefly, *Aleurodicus dispersus*. As important biological control agents against the spiralling whitefly, these members of *Nephaspis* have been introduced into different parts of the world, such as Hawaii, Guam, Fiji, Taiwan and Thailand (Waterhouse and Norris 1989, Chien et al. 2002, Napompeth 2004).

### Nephaspis indu﻿s

Gordon, 1996

Nephaspis indus Gordon, 1996: 43;Yu 2011: 88.
Nephaspis amnicola : Yoshida and Mau 1985 (nec. Wingo 1952​)

#### Materials

- **Type status:** Other material. **Occurrence:** catalogNumber: SCAU (E) 11661; recordedBy: Ren Shun-Xiang; individualCount: 8; sex: male; lifeStage: adult; **Taxon:** scientificName: Nephaspis indus; **Location:** island: Taiwan; locality: Zhiben, Taidong county; verbatimElevation: 140 m; locationRemarks: label transliteration: "Taiwan, Taidong, Zhiben, 2012.08.20, Ren Shunxiang"; [台湾台东知本 140 m, 22°41.63'N 120°59.94'E, 2012.08.20, sweeping, 任顺祥]; verbatimCoordinates: 22°41.63'N 120°59.94'E; decimalLatitude: 22.6938; decimalLongitude: 120.999; georeferenceProtocol: label; **Identification:** identifiedBy: Xiaosheng Chen; dateIdentified: 2016; **Event:** samplingProtocol: sweeping; eventDate: 2012-8-20; **Record Level:** modified: 2016-05-13T10:24:32Z; language: en; collectionCode: Insects; basisOfRecord: PreservedSpecimen
- **Type status:** Other material. **Occurrence:** catalogNumber: SCAU (E) 11670; recordedBy: Ren Shun-Xiang; individualCount: 7; sex: female; lifeStage: adult; **Taxon:** scientificName: Nephaspis indus; **Location:** island: Taiwan; locality: Zhiben, Taidong county; verbatimElevation: 140 m; locationRemarks: label transliteration: "Taiwan, Taidong, Zhiben, 2012.08.20, Ren Shunxiang"; [台湾台东知本 140 m, 22°41.63'N 120°59.94'E, 2012.08.21, sweeping, 任顺祥]; verbatimCoordinates: 22°41.63'N 120°59.94'E; decimalLatitude: 22.6938; decimalLongitude: 120.999; georeferenceProtocol: label; **Identification:** identifiedBy: Xiaosheng Chen; dateIdentified: 2016; **Event:** samplingProtocol: sweeping; eventDate: 2012-8-20; **Record Level:** modified: 2016-05-13T10:24:32Z; language: en; collectionCode: Insects; basisOfRecord: PreservedSpecimen

#### Description

TL: 1.23–1.32 mm, TW: 0.87–0.92mm, TH: 0.67–0.69mm, TL/TW: 1.41–1.43, PL/PW: 0.52–0.53, EL/EW: 1.05–1.07, HW/PW: 0.61–0.62, PW/EW: 0.84–0.85.

Body rounded oval, moderately convex, dorsum covered with white pubescence (Fig. 2a–f). Head, antennae and mouthparts yellow in male (Fig. 2a–c). Pronotum yellow with dark brown at central base. Scutellum black. Elytra black with apical 1/15 yellow. Prothoracic hypomeron and prosternum yellow. Mesoventrite and metaventrite reddish brown to black. Elytral epipleuron black. Legs yellow.

Head with fine frontal punctures, as large as eye facets, 0.5 diameter apart. Eyes densely faceted, interocular distance 0.44 times head width. Pronotal punctures smaller than those on frons, 1.0–1.5 diameters apart. Surface of elytra with punctures larger than those on pronotum, separated by 1.0–2.0 diameters. Prosternal process very short, transversely oval (Fig. 1b). Abdominal postcoxal lines strongly recurved and distinctly incomplete laterally (Fig. 2g), nearly reaching posterior margin of abdominal ventrite 1, area enclosed by lines coarsely punctate, broadly smooth along line. Abdominal ventrite 5 with apex truncate in both sexes.

Male genitalia. Penis long, strongly curved at basal 1/2 length (Fig. 2h); penis capsule with long inner arm and short outer arm; apex of penis with membranous appendage (Fig. 2i). Tegmen stout (Fig. 2j–k) with penis guide widest at base, tapering gradually to pointed apex in ventral view (Fig. 2j) and its base with prominent dorsal keel in lateral view (Fig. 2k). Parameres tapering toward apex, as long as 2/3 length of penis guide, covered with several long setae at apices (Fig. 2k).

Female externally similar to male but with black pronotum (Fig. 2d) except anterolateral angles yellow (Fig. 2e–f); head yellow with vertex dark brown (Fig. 2e).

#### Diagnosis

This species is similar to *Nephaspis bicolor* Gordon, 1982 in general appearance, but can be distinguished from the latter by details of male genitalia, particularly the stout penis guide with a dorsal keel at basal 1/3 length in lateral view (Fig. 2k) and the black pronotum with yellow anterolateral angles in female (Fig. 2e). In *N. bicolor*, the penis guide with a high dorsal keel at basal half in lateral view and the yellow pronotum with median 1/3 dark brown in female (Gordon 1996).

#### Distribution

Taiwan, Hawaii, Trinidad, Honduras.

#### Notes

There was some confusion about the taxonomy and nomenclature of *N. indus*. This species was introduced from Honduras, Trinidad and the West Indies into Hawaii as *N. amnicola* in 1979–1980 where it became effective in biological control of the spiralling whitefly (Kumashiro et al. 1983). A few years later, Gordon (1996) described a new species, *N. indus*, based on the specimens from Hawaii. Actually, this is a Trinidadian species, although the type series were all from Hawaii. He also pointed out that the male genitalia figured in Gordon (1982) for *N. bicolor* are those of *N. indus*, because a male of the latter species was mixed in with the type series of *N. bicolor* and that was the specimen selected for illustration (Gordon 1996).

## Supplementary Material

XML Treatment for Nephaspi﻿s﻿

XML Treatment for Nephaspis indu﻿s
